# Modifiable Risk Factors for Perinatal Depression among Women with Family History of Psychiatric Disorders: a nationwide register-based study in Sweden

**DOI:** 10.1192/j.eurpsy.2025.674

**Published:** 2025-08-26

**Authors:** R. Keijser, D. Lu

**Affiliations:** 1 Karolinska Institutet, Stockholm, Sweden

## Abstract

**Introduction:**

Perinatal depression (PND) is characterized by depressive episodes with an onset during pregnancy or following delivery. The clinical management of PND is however challenging because patients are often concerned of the potential harm of pharmaceutical therapy on foetus via placenta or breastfeeding. Previous findings have shown that women with a family history of psychiatric disorders (FhPsy) are at high risk for PND. However, no precision prevention strategy has been developed for this high-risk population.

**Objectives:**

To identify risk factors modifying the risk of perinatal depression (PND) among women with a family history of psychiatric disorders (FhPsy).

**Methods:**

We conducted a cohort study of 2,195,838 pregnancies of which 1,236,980 births between 2001-2021 in Sweden. PND was defined as a depression diagnosed, or antidepressant prescribed during pregnancy or within a year postpartum. FhPsy was assessed as any psychiatric disorder diagnosed before pregnancy among biological parents/siblings. Modifiable risk factors, i.e., snuff use and smoking before and during pregnancy, and BMI in early pregnancy and at delivery, were identified from registers. Multivariable logistic regression was used to estimate the OR of PND in relation to FhPsy, modifiable risk factors, and interaction effects.

**Results:**

In total, 94,597 (4.35%) women were diagnosed with PND at a mean age of 31.24 (SD = 5.30) years. Women with a FhPsy had 2.6 higher risk of PND (95% CI 2.58-2.61). All modifiable risk factors were also positive associated with PND (ORs ranged from 0.79 - 2.34). Interaction effects were observed between FhPsy and the modifiable factors (*P*-for-interaction <0.05). Specifically, women with FhPsy using snuff three months prior to pregnancy had 3.54 times higher odds of PND (95% CI 3.54-3.78), compared with women without such history who were non-snuff users. However, the OR was attenuated by 61% among those with FhPsy yet no use of snuff (OR 2.19, 95% CI 2.17-2.23). Similar trends were found for heavy-smoking-to-no-smoking either before or during early pregnancy and for obesity-to-normal-weight either during early pregnancy or at delivery among women with FhPsy, with an OR attenuation ranging from 11% to 58%.

**Conclusions:**

While women with a FhPsy are at risk for PND, modifying risk factors, i.e., smoking or snuff cessation and maintaining a healthy weight, may help lower such odds. Our findings have important implications for informing prevention strategies targeting the high-risk population.

**Disclosure of Interest:**

None Declared

